# Psychological Mechanisms Mediating Effects Between Trauma and Psychotic Symptoms: The Role of Affect Regulation, Intrusive Trauma Memory, Beliefs, and Depression

**DOI:** 10.1093/schbul/sbv175

**Published:** 2016-07-22

**Authors:** Amy Hardy, Richard Emsley, Daniel Freeman, Paul Bebbington, Philippa A. Garety, Elizabeth E. Kuipers, Graham Dunn, David Fowler

**Affiliations:** ^1^Department of Psychology, King’s College London, Institute of Psychiatry, Psychology and Neuroscience, London, UK;; ^2^Centre for Biostatistics, University of Manchester, Manchester, UK;; ^3^Department of Psychiatry, University of Oxford, Warneford Hospital, Oxford, UK;; ^4^UCL Mental Health Sciences Unit, University College London, London, UK;; ^5^NIHR Biomedical Research Centre for Mental Health, South London and Maudsley NHS Foundation Trust, London, UK;; ^6^School of Psychology, University of Sussex, Brighton, UK

**Keywords:** victimization, psychosis, reexperiencing, hyperarousal, avoidance, schema

## Abstract

Evidence suggests a causal role for trauma in psychosis, particularly for childhood victimization. However, the establishment of underlying trauma-related mechanisms would strengthen the causal argument. In a sample of people with relapsing psychosis (*n* = 228), we tested hypothesized mechanisms specifically related to impaired affect regulation, intrusive trauma memory, beliefs, and depression. The majority of participants (74.1%) reported victimization trauma, and a fifth (21.5%) met symptomatic criteria for Posttraumatic Stress Disorder. We found a specific link between childhood sexual abuse and auditory hallucinations (adjusted OR = 2.21, SE = 0.74, *P* = .018). This relationship was mediated by posttraumatic avoidance and numbing (OR = 1.48, SE = 0.19, *P* = .038) and hyperarousal (OR = 1.44, SE = 0.18, *P* = .045), but not intrusive trauma memory, negative beliefs or depression. In contrast, childhood emotional abuse was specifically associated with delusions, both persecutory (adjusted OR = 2.21, SE = 0.68, *P* = .009) and referential (adjusted OR = 2.43, SE = 0.74, *P* = .004). The link with persecutory delusions was mediated by negative-other beliefs (OR = 1.36, SE = 0.14, *P* = .024), but not posttraumatic stress symptoms, negative-self beliefs, or depression. There was no evidence of mediation for referential delusions. No relationships were identified between childhood physical abuse and psychosis. The findings underline the role of cognitive-affective processes in the relationship between trauma and symptoms, and the importance of assessing and treating victimization and its psychological consequences in people with psychosis.

## Introduction

There is increasing evidence suggesting that the aetiology of psychosis is to a large extent psychosocial.^1,2^ Putative social causes include childhood victimization trauma.^3,4^ While the demonstration of causal relationships can never be completely validated, there are a number of strategies that together increase their likely veracity.^5^ In the context of a supposed trauma-psychosis link, these include a clear temporal sequence between cause and consequence, the robust replication of an association, the existence of a dose-response curve, and the demonstration of plausible and theoretically-based mechanisms. The resolution of trauma-related consequences should also result in symptom reduction.

In most psychosis research, trauma accounts are obtained from individuals some time after the events occurred, and, therefore, rely on recall being not distorted. In case-control studies, trauma histories are elicited from cases after the onset of psychosis, adding to the problems of interpretation. However, the accounts of people with psychosis are reasonably reliable^6,7^ and case-control investigations have been corroborated by prospective cohort studies.^8^ The association between childhood victimization and psychosis appears consistently strong: in methodologically robust epidemiological surveys, prospective studies, and case-control studies, it has been replicated both in relation to diagnosed psychosis and to psychotic symptoms in nonclinical samples; it also shows a clear dose-response relationship.^3,4,9–11^ The ending of events has also been associated with symptom reduction in a nonclinical sample.^12^

Evidence for specific associations between trauma and symptom type adds further support for a causal link, in that particular events may be more likely to trigger specific psychological mechanisms. Findings from general population and clinical samples suggest specific associations (with ORs of >2 in most studies) between childhood sexual abuse with voices, of childhood physical abuse and childhood neglect with paranoia, and of parental communication deviance with formal thought disorder.^13^

However, there have been relatively few investigations of trauma-related psychological mechanisms in clinical samples. The current study aims to replicate previous research linking trauma and psychotic symptoms and then to test theory-based hypotheses about potential causal mechanisms in a clinical sample of people with relapsing psychosis. Three such mechanisms have recently been put forward as potential causal paths linking trauma to psychosis.

The first involves the ways in which people regulate affect or threat. Read et al^14^ propose exposure to childhood victimization results in neurodevelopmental changes such as hyperactivity of the hypothalamic-pituitary axis. This leaves people vulnerable to understandable but unhelpful ways of regulating stress, which may then contribute to psychosis. We respond to threat with varying degrees of sympathetic (ie, fight and flight) and parasympathetic (ie, flag and faint) nervous system activation (the “defense cascade”), depending on the nature of the event and the individual.^15^ Once aroused, avoidant or detached dissociative responses may be protective, but they can become habitual and paradoxically perpetuate perceptions of threat. The role of affect regulation in psychosis has been demonstrated in studies showing that threat-biased information processing, dissociation, and avoidance are associated with symptom severity.^16–19^ The current study will test the hypothesis that trauma-related affect regulation (as assessed by posttraumatic avoidance and numbing, and hyperarousal symptoms) mediates identified associations between trauma, hallucinations, and delusions.

Second, we will examine the role of intrusive memory in the link between trauma and hallucinations. Memory encoding and retrieval is disrupted by arousal and dissociative detachment during trauma, such that we are more likely to store and remember sensory-perceptual than contextual information.^20^ This results in involuntary memory intrusions and impaired intentional recall. Evidence suggests that, following trauma, people with a vulnerability to psychosis may be more susceptible to experiencing intrusions lacking in contextual information, potentially resulting in more frequent, and fragmented images.^21^ Findings indicate intrusive trauma memory is associated with hallucinations, and has been implicated in the relationship between childhood sexual abuse and hallucinations.^18,22,23^ We therefore hypothesize that intrusive trauma memory will mediate the identified association between childhood sexual abuse and hallucinations.

The final mechanism concerns the role of trauma-related beliefs in the link between trauma and paranoia.^24^ These appraisals reflect cognitive representations of the self and others, or internal working models.^25^ Trauma is associated with negative beliefs about the self and others in psychosis, and this, at least partly, has accounted for its relationship with paranoia in clinical and nonclinical samples.^26–28^ Therefore, we predict negative beliefs will account for the identified relationship between childhood physical abuse and paranoia. Emotion is likely to be involved in all of the proposed causal mechanisms, given its well-established role as a precursor to, component of, and consequence of psychosis and trauma.^29,30^ The impact of depression on identified trauma and symptom associations will therefore be investigated.

In summary, we aim to investigate the psychological mechanisms involved in associations between trauma type and specific symptoms in people with relapsing psychosis. The mechanisms considered include affect regulation, intrusive trauma memory, beliefs, and depression. We hypothesize (1) that childhood sexual abuse and auditory hallucinations, and childhood physical abuse and paranoia will be linked independently of demographic variables; (2) that intrusive trauma memory will mediate the relationship between childhood sexual abuse and hallucinations; (3) that negative self and other beliefs will mediate associations between childhood physical abuse and paranoia; and (4) that affect and affect regulation processes (posttraumatic avoidance and numbing and hyperarousal symptoms, and depression) will mediate all identified relationships between trauma and psychosis.

## Method

The sample was recruited for the Psychological Prevention of Relapse in Psychosis (PRP) Trial (ISRCTN83557988), a UK multicenter randomized controlled trial of cognitive behavioral therapy and family intervention for psychosis, based in 4 NHS Trusts in London and East Anglia.^31^ The study protocol was designed a priori to address questions about psychosocial processes associated with psychosis outcomes.

### Participants

The sample comprised individuals with psychosis who had experienced a recent relapse in positive symptoms, either from a previously recovered state or from a state of persisting symptoms. They were recruited from inpatient and outpatient services. The inclusion criteria were: a current diagnosis of nonaffective psychosis, age between 18 and 65 years, a second or subsequent episode starting not more than 3 months before consent, and a rating of at least 4 (moderate severity) on the delusions, hallucinations, grandiosity, or suspiciousness/persecution items of the Positive and Negative Syndrome Scale (PANSS).^32^ The exclusion criteria were: a primary diagnosis of substance misuse, organic syndrome or learning disability, residential instability, and insufficient command of English to engage in therapy.

After providing informed consent, participants completed assessments of symptoms and psychosocial processes at baseline, and at 3-, 6-, 12-, and 24-month follow-up. Trauma and posttraumatic stress data for this study were collected as part of the trial assessment battery at 3-month follow-up, and symptom and mechanism data were therefore also taken from this time-point.

### Measures

#### The Trauma History Questionnaire.

The Trauma History Questionnaire (THQ)^33^ is a structured interview for assessment of nonvictimization and victimization exposure. Acceptable psychometrics have been established for the THQ in psychosis samples.^34,35^ If participants reported at least 1 event, they were asked to indicate which trauma they were currently most affected by (categorized as the “index” event). This could include discrete, episodic, or persistent events. All reported events were categorized according to trauma type by childhood, adulthood, and lifetime prevalence. Trauma type was categorized into nonvictimization (ie, illness, accidents, and natural disasters) and victimization events. Victimization trauma was categorized into sexual, physical, and emotional abuse. Sexual abuse was coded from 2 items pertaining to child abuse (under age 13 and under age 18), and the adult abuse item. Physical abuse was coded from 2 items assessing physical attack (with and without a weapon) and the bullying item, which was reviewed for reference to interpersonal violence. Emotional abuse was categorized from reports of psychological abuse in the bullying item. Finally, event descriptions for the remaining items were examined for reports of victimization and coded accordingly.

#### The Scales for the Assessment of Positive Symptoms.

The Scales for the Assessment of Positive Symptoms^36^ contains 35 items measuring positive symptoms of psychosis and is rated on a 6-point scale (0–5) over the past month. For this study, symptoms were defined as absent (0 and 1) or present (2–5). Auditory hallucinations were coded from the auditory hallucinations, voices commenting and voices conversing items. The persecutory and referential delusions items were used to code these symptoms.

#### Self-report Scale for Posttraumatic Stress Disorder.

Participants were asked to complete the Self-report Scale for Posttraumatic Stress Disorder (SRS-PTSD)^37^ in relation to any identified index event. It consists of 17 items each corresponding to a diagnostic criterion of PTSD in DSM-IV.^38^ There are 5 reexperiencing or intrusive trauma memory, 7 avoidance and emotional numbing, and 5 hyperarousal items. Each item is rated on a 3-point scale from 0 to 2, assessed over the past month. PTSD symptom criteria are met in the presence of 1 reexperiencing, 3 avoidance, and 2 hyperarousal items. Internal consistency (Cronbach’s alphas of >.87), interrater reliability (kappa = 0.98) and likelihood ratios for positive and negative test results (4.3 and 0.18, respectively) are reported.

#### Brief Core Schema Scale.

The Brief Core Schema Scale (BCSS)^39^ is a 24-item self-report questionnaire rated on a 5-point scale (0–4) for the assessment of core beliefs. The negative-self and negative-other scales were used for the purpose of this study. The BCSS shows good internal consistency and Cronbach’s alpha coefficients are >.78.

#### Beck Depression Inventory II.

The Beck Depression Inventory (BDI-II) is a self-report questionnaire comprising 21 items, each rated on a 4 point scale (0–3) providing a total score (0–63).^40^ Depression is assessed over the past fortnight.

## Analysis

All analyses were conducted using Stata v13.1.^41^ For the purposes of clarity and comparison with previous results, we excluded any psychosis-related events based on hallucinations and delusional appraisals that were reported in response to the THQ event type prompts. We used summary statistics (means and SD, number and percentage) to describe the sample and display the prevalence, putative mediators, and outcomes. First we assessed bivariate associations between all trauma types and symptoms. Then, for any significant associations, we performed logistic regression to assess the total effect of exposure on outcome adjusting for age, gender, and ethnicity as potential confounders. To assess the effect of exposure on putative mediators, we performed linear regression adjusting for the same set of confounders. Only those trauma-outcome and trauma-mediator relationships which were statistically significant at the 10% level were carried forward into the mediation analysis.

The mediating role of trauma-related psychological mechanisms in any identified associations between events and symptoms was examined using the counterfactual framework as summarized in Valeri and VanderWeele^42^ and implemented with the –paramed- command in Stata.^41^ We estimate the natural direct effect (NDE), which expresses how much the outcome would change in the presence vs absence of trauma, but for each individual if the mediator was kept at the level it would be if trauma had not occurred. We assume no interaction between exposure and mediator, implying the NDE is the same as the controlled direct effect. The corresponding natural indirect effect (NIE) is defined as how much the outcome would change on average if trauma was present and the mediator changed from the level it would be in the absence of trauma to the level it would be if trauma occurred. For a binary outcome, the total effect is the product between natural direct and natural indirect effects.

To estimate these causal effects, first the effect of trauma type on mediator was estimated using linear regression. Second, the effects of trauma type and the mediator on outcome were estimated using logistic regression. Both models also adjust for baseline demographics (age, sex and ethnicity). The relevant parameters from these models are combined to estimate the NDE and NIE, with SEs obtained using the delta method. A complete case analysis is conducted for each set of trauma-mediator-outcome-covariates, with the size of each analysis sets reported. Mediators were considered to reflect a partial indirect effect if the NIE was statistically significant, and evidence of full mediation if the direct effect also became nonsignificant.

## Results

### Sample

The PRP sample consisted of 301 participants. Two hundred twenty-eight (76% of the total) completed the THQ and were included in the current study. From a possible 3420 events (*n* = 228 participants reporting on 15 event types), only 28 events (0.82%) were reported that appeared to be the consequence of hallucinatory or delusional experience. The participants who completed the trauma measures were compared with those who did not on a range of demographic and clinical variables (ie, age, length of illness, gender, ethnicity, marital status, employment, and risk). There were no significant differences between the groups on any of the variables, indicating the trauma sample was sufficiently representative (*t* = −0.39–1.70, *P* = .090–.693, χ^2^ = −0.49–8.44, *P* = .134–.740). The mean age of participants was 38.24 years (SD = 11.11). There were more male (*n* = 165) than female (*n* = 63) participants. The majority of the sample were White (*n* = 167), then Black African (*n* = 23), Black Caribbean (*n* = 17) and other (*n* = 21). Most were single (*n* = 167), unemployed (*n* = 182) and inpatients at the time of recruitment into the trial (*n* = 155). Diagnoses were schizophrenia (*n* = 195), schizo-affective disorder (*n* = 29), and delusional disorder (*n* = 4). The mean length of contact with mental health services was 10.83 years (SD = 9.06).

### Trauma

The rates of trauma types reported by the sample are shown in table 1. The majority of the sample reported experiencing a lifetime trauma (86.0%), with most reporting at least 1 lifetime victimization event (74.1%) and two-thirds reporting a lifetime nonvictimization event (66.2%). The mean number of traumatic events reported was 2.92 (SD = 2.12, range 0–10). A fifth of the sample reported childhood sexual abuse or childhood physical abuse, and a third described experiencing childhood emotional abuse. Just under a fifth of the sample reported adulthood sexual abuse or adulthood emotional abuse, whereas 40% indicated experiences of adulthood physical abuse. Overall, approximately half of the sample experienced lifetime physical abuse and lifetime emotional abuse, and just under a third lifetime sexual abuse. The rates of individual traumatic events, index traumas, and symptom criteria for PTSD are shown in table 2. The most common events were bullying, road traffic accidents then physical attacks. One hundred twenty-four participants (54.4%) reported an index trauma (ie, an event they were still currently affected by), with bullying, other stressful events, and physical attacks being most frequent. Symptom criteria for PTSD were met by 49 participants (21.5%), and were most likely to be associated with bullying, physical attacks then childhood sexual abuse.

**Table 1.** Rates of Trauma and Abuse Type (*N* = 228)

Trauma Category			*n*	%
Trauma type	All	Child	142	62.3
		Adult	172	75.4
		Lifetime	196	86.0
	Nonvictimization	Child	78	34.2
		Adult	122	53.5
		Lifetime	151	66.2
	Victimization	Child	114	50.5
		Adult	124	54.4
		Lifetime	169	74.1
Abuse type	Sexual	Child	50	21.9
		Adult	35	15.4
		Lifetime	69	30.3
	Physical	Child	50	21.9
		Adult	95	41.7
		Lifetime	108	47.4
	Emotional	Child	72	31.6
		Adult	35	15.4
		Lifetime	99	43.2

**Table 2.** Traumatic Event (*N* = 228), Index Event (*n* = 143) and Posttraumatic Stress Disorder (PTSD; *n* = 49) Prevalence by Event Type

Event Type	Prevalence		Index		PTSD	
	*n*	%	*n*	%	*n*	%
War	7	3.1	1	1.3	0	0.0
Traffic accident	80	35.1	10	12.4	3	6.1
Natural disaster	22	9.7	0	0.0	0	0.0
Serious illness	23	10.1	3	3.7	1	2.0
Childhood sexual abuse: <age 13	26	11.6	5	6.2	2	4.1
Childhood sexual abuse: >age 12 and <age 18	30	13.4	9	11.2	6	12.2
Adulthood sexual abuse	32	14.4	9	11.2	4	8.2
Physical attack with weapon	64	28.3	12	14.9	4	8.2
Physical attack no weapon	70	31.3	14	17.4	6	12.2
Other events with actual serious injury	46	20.3	1	1.2	0	0.0
Other events with threat of injury/death	40	17.8	1	1.3	0	0.0
Witnessing serious harm or death	51	22.5	2	2.6	1	2.0
Other stressful events	55	24.3	16	19.8	3	6.1
Relative or friend killed or injured	19	8.4	6	7.4	3	6.1
Bullying	99	44.8	35	43.4	16	32.7

### Trauma and Symptoms

The associations between trauma types and psychotic symptoms are shown in table 3. Consistent with our first hypothesis, there was a significant association between childhood sexual abuse and auditory hallucinations. However, our second hypothesis was not supported, as there was no association between childhood physical abuse and paranoia. In contrast, childhood emotional abuse was associated with persecutory beliefs and delusions of reference. These associations remained significant after adjusting for age, gender, and ethnicity, with OR ranging from 2.21 to 2.43 (supplementary table 1). No other significant associations between trauma type, auditory hallucinations, persecutory, and referential delusions were identified. There were also no significant effects of combined trauma types or cumulative trauma on symptoms (supplementary table 2).

**Table 3.** Bivariate Associations Between Trauma Type and Symptoms (*N* = 228)

		Auditory							Persecutory							Referential						
		Absent		Present					Absent		Present					Absent		Present				
		*n*	%	*N*	%	χ^2^	*P*	OR (CI)	*n*	%	*n*	%	χ^2^	*P*	OR (CI)	*n*	%	*n*	%	χ^2^	*P*	OR (CI)
CNV^a^	No	87	59	60	41	2.26	.133	1.5 (0.9–2.7)	78	53	69	47	0.97	.324	1.3 (0.8–2.3)	91	62	56	38	.378	.539	1.2 (0.7–2.1)
	Yes	38	49	40	51				36	46	42	54				45	58	33	42			
ANV^b^	No	58	55	47	45	0.01	.929	0.8(0.6–1.7)	53	51	52	49	0.01	.957	1.0(0.6–1.7)	63	60	42	40	0.02	.899	1.0 (0.6–1.7)
	Yes	67	56	53	44				61	51	59	49				73	61	47	39			
CSA^c^	No	105	60	70	40	6.30	.012*	2.3 (1.2–4.3)	89	51	86	50	0.01	.915	1.0 (0.6–1.9)	108	62	67	38	0.53	.466	1.3 (0.7–2.4)
	Yes	20	40	30	60				25	50	25	50				28	56	22	44			
ASA^d^	No	107	56	84	44	0.11	.739	1.1 (0.5–2.4)	97	51	94	49	0.01	.933	1.0 (0.5–2.1)	115	60	76	40	0.03	.864	0.9 (0.4–2.0)
	Yes	18	53	16	47				17	50	17	50				21	62	13	38			
CPA^e^	No	97	55	79	45	0.07	.800	0.9 (0.5–1.7)	91	52	85	48	0.35	.555	1.2 (0.6–2.3)	110	63	66	38	1.43	.232	1.5 (0.8–2.8)
	Yes	28	57	21	43				23	47	26	53				26	53	23	47			
APA^f^	No	74	56	58	44	0.03	.856	1.1 (0.6–1.8)	68	52	64	48	0.09	.762	1.1 (0.6–1.8)	82	62	50	38	0.38	.540	1.2 (0.7–2.0)
	Yes	51	55	42	45				46	49	47	51				54	58	39	42			
CEA^g^	No	87	57	67	44	0.18	.677	1.1 (0.6–2.0)	86	56	68	44	5.23	.022*	1.9 (1.1–3.5)	102	66	52	34	6.84	.009**	2.1 (1.2–3.8)
	Yes	38	54	33	47				28	39	43	61				34	48	37	52			
AEA^h^	No	106	56	84	44	0.03	.869	1.1 (0.5–2.2)	98	52	92	48	0.41	.524	1.3 (0.6–2.6)	117	62	73	38	0.66	.417	1.4 (0.7–2.8)
	Yes	19	54	16	46				16	46	19	54				19	54	16	46			

*Note*: ^a^Child nonvictimization.

^b^Adult nonvictimization.

^c^Child sexual abuse.

^d^Adult sexual abuse.

^e^Child physical abuse.

^f^Adult physical abuse.

^g^Child emotional abuse.

^h^Adult emotional abuse.

*P < .05.; **P < .01.

### Trauma and Hypothesized Trauma-Related Mediators

The relationships between trauma types and trauma-related psychological mechanisms are shown in table 4. In line with our hypotheses, childhood sexual abuse was associated with posttraumatic numbing and avoidance, and hyperarousal, but had only a weak association with intrusive trauma memory (*P* = .107). In addition, childhood sexual abuse, childhood physical abuse, and childhood emotional abuse were associated with more severe negative-other beliefs. None of the trauma types were significantly associated with depression and negative-self beliefs. In supplementary table 3, the effects of combined trauma types on the mediators are shown. The presence of both childhood sexual abuse and childhood emotional abuse was associated with more severe negative-other beliefs and posttraumatic hyperarousal than the individual event types. The combination of childhood sexual abuse and childhood emotional abuse was not associated with more severe intrusive trauma memory or posttraumatic avoidance and numbing.

**Table 4.** Means (M), Standard Deviations (SD), Adjusted Mean Difference (aMD), and Statistical Significance (*P*) Between Trauma Type and Mediators

		Intrusive Trauma Memory^a^			Posttraumatic Numbing and Avoidance^a^			Posttraumatic Hyperarousal^a^			Negative-Other^b^			Negative-Self^c^			Depression^d^		
		*M*	SD	aMD (SE), *P*	*M*	SD	aMD (SE), *P*	*M*	SD	aMD (SE), *P*	*M*	SD	aMD (SE), *P*	*M*	SD	aMD (SE), *P*	*M*	SD	aMD (SE), *P*
CSA	No	4.55	3.52	1.18 (0.73), .107	6.61	4.17	2.49 (0.90), .006	4.37	3.38	1.81 (0.70), .011	7.52	7.12	2.85 (1.34), .035	5.63	5.38	1.36 (1.03), .190	19.37	12.74	2.62 (2.21), .237
	Yes	5.94	3.42		9.06	4.23		6.26	3.43		10.10	8.52		6.92	6.91		22.11	13.05	
CPA	No	4.70	3.63	0.97 (0.73), .187	7.24	4.46	0.16 (0.93), .861	4.72	3.61	0.79 (0.72), .276	7.53	7.15	2.53 (1.29), .051	5.61	5.56	1.48 (0.99), .137	19.64	12.98	1.86 (2.16), .392
	Yes	5.53	3.21		7.30	3.90		5.30	3.08		9.86	8.40		6.93	6.24		21.02	12.30	
CEA	No	4.59	3.82	0.94 (0.66), .155	7.19	4.54	0.18 (0.83), .832	4.68	3.60	0.58 (0.65), .374	7.17	7.04	3.09 (1.15), .008	5.49	5.64	1.42 (0.89), .111	19.61	12.90	1.55 (1.93), .423
	Yes	5.45	2.95		7.36	3.93		5.18	3.29		10.03	8.11		6.78	5.85		20.70	12.71	

*Note*: ^a^n = 118.

^b^n = 190.

^c^n = 194.

^d^n = 201.

### Trauma, Symptoms, and Mechanisms

Given the relationships identified, we next tested our hypothesis that intrusive trauma memory and affect regulation processes would mediate the relationship between childhood sexual abuse and hallucinations. Mediation by negative-other beliefs was also investigated. The mediation hypothesis for childhood physical abuse was not examined, as this event type was not associated with persecutory beliefs. Similarly, mediation by negative-self beliefs and depression was not investigated, as these processes were not associated with trauma in our sample. However, we did examine whether negative-other beliefs mediated the relationships between childhood emotional abuse, persecutory beliefs and delusions of reference.

Table 5 shows the results of the mediation analysis. The total effect of childhood sexual abuse on auditory hallucinations had an OR of 2.929 (SE = 0.479, *P* = .025), which decomposed into a natural direct effect of 2.438 (SE = 0.465, *P* = .055) and a natural indirect effect of 1.201 (SE = 0.133, *P* = .169). The indirect effect was not statistically significant, indicating that there was no mediation through intrusive trauma memory.

**Table 5.** Total, Direct, and Indirect Effects

Trauma Type	Symptom	Mediator	Total OR (SE), *P*	Direct Effect OR (SE), *P*	Indirect Effect OR (SE), *P*	n
CSA	Auditory hallucinations	Intrusive trauma memory	2.929 (0.479), .025	2.438 (0.465), .055	1.201 (0.133), .169	118
CSA	Auditory hallucinations	Posttraumatic avoidance & numbing	3.026 (0.493), .025	2.052 (0.475), .131	1.475 (0.188), .038	118
CSA	Auditory hallucinations	Posttraumatic hyperarousal	3.027 (0.494), .025	2.104 (0.477), .119	1.439 (0.184), .048	118
CSA	Auditory hallucinations	Negative other beliefs	2.763 (0.395), .010	2.343 (0.387), .028	1.179 (0.098), .094	190
CEA	Persecutory delusions	Negative other beliefs	2.568 (0.376), .012	1.889 (0.356), .074	1.359 (0.136), .024	190
CEA	Delusions of reference	Negative other beliefs	2.303 (0.345), .016	1.950 (0.342), .051	1.181 (0.091), .069	190

*Note*: Results show OR, SE, and statistical significance (*P*).

The indirect effect of childhood sexual abuse on auditory hallucinations through posttraumatic avoidance and numbing (OR = 1.475, SE = 0.188, *P* = .038) was significant, and the direct effect was nonsignificant (OR = 2.052, *P* = .131) indicating mediation. The same pattern of results was observed for a significant indirect effect through posttraumatic hyperarousal (indirect effect OR = 1.439, SE = 0.184, *P* = .045).

There was no evidence of mediation through negative-other beliefs for the total effect of childhood sexual abuse on auditory hallucinations, or for the total effect of childhood emotional abuse on referential delusions. There was a significant indirect effect of childhood emotional abuse on persecutory delusions through negative-other beliefs (OR = 1.359, SE = 0.136, *P* = .024), which decomposes the total effect (OR = 2.568, *P* = .012) and the direct effect became nonsignificant (OR = 1.889, *P* = .074).

## Discussion

This study is the first to demonstrate that trauma-related psychological mechanisms mediate victimization and psychotic symptoms associations in a large sample of people with relapsing psychosis. The identification of theoretically based psychological processes underlying specific associations between events and symptoms provides further support for the causal role of trauma in psychosis. Consistent with our hypothesis, childhood sexual abuse was associated with auditory hallucinations. The mediation hypotheses were partially supported, as posttraumatic avoidance, numbing, and hyperarousal (but not intrusive trauma memory or depression) accounted for this relationship. We did not find the hypothesized link between childhood physical abuse and paranoia, although persecutory and referential delusions were related to childhood emotional abuse. This suggests it may be psychological rather than physical threat in interpersonal relationships that is critical to the maintenance of paranoia in psychosis. Negative-other beliefs accounted for the relationship between childhood emotional abuse and persecutory delusions, and depression and posttraumatic affect regulation did not play a role.

As expected, we found a higher rate of trauma, particularly victimization trauma than in the general population.^43^ The rates of childhood sexual abuse and childhood emotional abuse identified are comparable to those reported in Bonoldi and colleagues’^44^ meta-analysis, although the rate of childhood physical abuse in this study was somewhat lower. It is of note that the victimization rates we identified are lower than earlier studies of trauma prevalence in psychosis.^45^ This may be attributable to more robust assessment, a higher false negative rate or reducing the sampling bias that may occur when participants are recruited to studies solely focused on investigating trauma. Symptomatic criteria for PTSD was met in a fifth of the sample using a self-report measure, which is comparable to the rate reported (16%) in a recent study employing a gold-standard interview assessment.^46^

Our findings are consistent with cognitive-behavioral models of psychosis that highlight the causal role of cognitive-affective processes in symptom development and maintenance.^25,31^ The specificity of associations found between particular trauma types and symptoms further supports this argument.^14^ While other researchers have argued childhood victimization has a nonspecific effect on psychosis, these findings relate to samples with less severe psychotic symptoms that may be less sensitive to detecting specific effects.^12,47,48^ However, investigating the associations between trauma and symptom is complex, given that people often experience multiple event and symptom types. Our results suggest a role for posttraumatic affect regulation processes in hallucinations, and negative beliefs about others in paranoia, and support theoretical models regarding the developmental impact of trauma on psychosis.^15,25,26^ Garety and Freeman^49^ argue talking therapies should target underlying causal mechanisms to improve their modest effects on psychotic symptoms. These findings indicate interventions modifying trauma-related affect regulation and beliefs may have benefit, such as coping strategy enhancement techniques, and verbal and experiential cognitive restructuring.^50^

Contrary to our expectations, we found no evidence of mediation by intrusive trauma memory, negative-self beliefs or depression. However, the study was a stringent test of these hypotheses as our sample had relatively high rates of depression and negative beliefs.^51^ In relation to trauma memory, a significant limitation was that intrusive memories were assessed in relation to the index event; this was often not sexual abuse, and so intrusions related to this event type may have been missed. However, it is also possible that trauma memories are decontextualized in people with persistent psychosis such that intrusions are experienced as hallucinations and not reexperiencing of the event.^22^ These would be maintained by understandable, but maladaptive, affect regulation strategies such as hyperarousal, avoidance, and numbing. Promising findings have already been reported for trauma-focused exposure treatments aiming to contextualize and elaborate trauma memories in psychosis, and the impact of these interventions on psychotic symptom severity should be investigated.^52^

Other limitations of the findings are that there was no direct assessment of neglect and psychosis-related trauma. Given the mediating role of posttraumatic stress numbing symptoms and the well-established role of dissociation in psychosis, it would have been useful to assess dissociative detachment symptoms, including depersonalization and derealisation.^53^ Another aspect of affect regulation that appears worthy of consideration in the trauma and psychosis association is affective dysregulation.^54^ Our findings suggest oscillations between arousal and numbing may drive hallucinatory experience in people with psychosis. The trauma measure also provided limited information on the severity of physical and psychological harm. This may account for the lack of a dose-response relationship between events and symptoms, or an impact of cumulative trauma. The study was cross-sectional and future work should investigate these processes in prospective or longitudinal studies, using comprehensive interview assessments of posttraumatic mechanisms. Further exploration of these mechanisms in clinical groups will inform the development of trauma-focused talking treatment for psychosis.

In conclusion, the findings suggest trauma-related psychological mechanisms mediate the specific associations between victimization and psychotic symptoms in a sample of people with relapsing psychosis. The study supports the growing call for mental health care providers to tailor psychosis services to the specific needs of people affected by trauma, including assessment and treatment of victimization and its psychological consequences.^55^

## Supplementary Material

Supplementary material is available at http://schizophreniabulletin.oxfordjournals.org.

## Funding

This work was supported by the Wellcome Trust (062452).

## Supplementary Material

Supplementary Data
