# Supplementary material for: Psychological Mechanisms Mediating Effects Between Trauma and Psychotic Symptoms: The Role of Affect Regulation, Intrusive Trauma Memory, Beliefs, and Depression
Source: Schizophr Bull. 2016 Jul 22;42(Suppl 1):S34–43. doi: 10.1093/schbul/sbv175 (PMC4960432; doi:10.1093/schbul/sbv175)
Supplement: Supplementary Data [file supp_42_suppl-1_S34__index.html]

Psychological Mechanisms Mediating Effects Between Trauma and Psychotic Symptoms: The Role of Affect Regulation, Intrusive Trauma Memory, Beliefs, and Depression — Supplementary Data 

# Psychological Mechanisms Mediating Effects Between Trauma and Psychotic Symptoms: The Role of Affect Regulation, Intrusive Trauma Memory, Beliefs, and Depression

## Supplementary Data

Data files

- Supplementary Data - Supplementary Data
