# Supplementary material for: Psychological Mechanisms Mediating Effects Between Trauma and Psychotic Symptoms: The Role of Affect Regulation, Intrusive Trauma Memory, Beliefs, and Depression
Source: Schizophr Bull. 2016 Jul 22;42(Suppl 1):S34–43. doi: 10.1093/schbul/sbv175 (PMC4960432; doi:10.1093/schbul/sbv175)
Supplement: Supplementary Data [file supp_sbv175_SupplementarymaterialPsychologicalMediatorsofTraumaandPsychosisHardyetal3.doc]

Supplementary material

- Table 4: Association between trauma type and symptoms controlling for age, gender and ethnicity. Results show adjusted odds ratio (aOR), standard errors (SE) and statistical significance (p). N=228.

|  | | - Auditory | | | - Persecutory | | | - Referential | | |
| --- | --- | --- | --- | --- | --- | --- | --- | --- | --- | --- |
|  |  | - Absent | - Present |  | - Absent | - Present |  | - Absent | - Present |  |
|  |  | - n (%) | - n (%) | - aOR (SE), p | - n (%) | - n (%) | - aOR (SE), p | - n (%) | - n (%) | - aOR (SE), p |
| - CSA^a^ | - No | - 105 (60) | - 70 (40) | - 2.21 (0.74), - 0.018 | - 89 (51) | - 86 (50) | - 1.06 (0.35), - 0.866 | - 108 (62) | - 67 (38) | - 1.30 (0.44), - 0.433 |
|  | - Yes | - 20 (40) | - 30 (60) |  | - 25 (50) | - 25 (50) |  | - 28 (56) | - 22 (44) |  |
| - CPA^b^ | - No | - 97 (55) | - 79 (45) | - 0.92 (0.30), - 0.805 | - 91 (52) | - 85 (48) | - 1.35 (0.45), - 0.369 | - 110 (63) | - 66 (38) | - 1.65 (0.56), - 0.139 |
|  | - Yes | - 28 (57) | - 21 (43) |  | - 23 (47) | - 26 (53) |  | - 26 (53) | - 23 (47) |  |
| - CEA^c^ | - No | - 87 (57) | - 67 (43) | - 1.15 (0.34), - 0.627 | - 86 (56) | - 68 (44) | - 2.21 (0.68), - 0.009 | - 102 (66) | - 52 (34) | - 2.43 (0.74), - 0.004 |
|  | - Yes | - 38 (54) | - 33 (46) |  | - 28 (39) | - 43 (61) |  | - 34 (48) | - 37 (52) |  |

- ^a^ Child sexual abuse ^b^ Child physical abuse ^c^ Child emotional abuse
- Supplementary material
- Table 5. Association between multiple trauma type and symptoms. Results show odds ratio (OR), standard errors (SE) and statistical significance (p) relative to the reference category.

| - Type of abuse | - n | - Auditory | | - Persecutory | |  | - Referential |
| --- | --- | --- | --- | --- | --- | --- | --- |
|  |  | Symptom present   - n (%) | OR (SE), p | Symptom present   - n (%) | - OR (SE), p | Symptom present   - n (%) | - OR (SE), p |
| - Neither | - 129 | - 48 (37) | - 1 | - 56 (44) | - 1 | - 41 (32) | - 1 |
| - CEA^a^ | - 49 | - 22 (45) | - 1.50 (0.52), 0.244 | - 29 (62) | - 2.46 (0.90), 0.014 | - 26 (53) | - 3.18 (1.18), 0.002 |
| - CSA^b^ | - 28 | - 19 (68) | - 3.46 (1.57), 0.006 | - 13 (46) | - 1.20 (0.52), 0.681 | 11 (39) | - 1.49 (0.67), 0.377 |
| - CEA^a^ + CSA^b^ | - 22 | - 11 (50) | - 1.49 (0.68), 0.384 | - 13 (59) | - 1.75 (0.82), 0.238 | - 11 (50) | - 2.05 (0.97), 0.130 |
| - Neither | - 144 | - 57 (40) | - 1 | - 68 (48) | - 1 | - 49 (35) | - 1 |
| - CPA^c^ | - 34 | - 12 (36) | - 0.84 (0.34), 0.676 | - 17 (52) | - 1.25 (0.50), 0.576 | - 17 (52) | - 2.25 (0.92), 0.046 |
| - CSA^b^ | - 34 | - 21 (62) | - 2.32 (0.92), 0.034 | - 16 (47) | - 0.95 (0.37), 0.889 | - 16 (47) | - 1.69 (0.67), 0.189 |
| - CPA^c^ + CSA^b^ | - 16 | - 9 (56) | - 1.88 (1.02), 0.243 | - 9 (56) | - 1.59 (0.87), 0.399 | - 6 (38) | - 1.27 (0.72), 0.669 |
| - Neither | - 135 | - 59 (44) | - 1 | - 57 (43) | - 1 | - 41 (31) | - 1 |
| - CPA^c^ | - 21 | - 8 (38) | - 0.79 (0.38), 0.624 | - 11 (52) | - 1.62 (0.79), 0.328 | - 11 (52) | - 2.81 (1.39), 0.038 |
| - CEA^a^ | - 43 | - 20 (47) | - 1.13 (0.40), 0.730 | - 28 (65) | - 2.82 (1.08), 0.007 | - 25 (58) | - 3.59 (1.37), 0.001 |
| - CPA^c^ + CEA^a^ | - 29 | - 13 (46) | - 1.09 (0.46), 0.836 | - 15 (54) | - 1.85 (0.80), 0.157 | - 12 (43) | - 2.01 (0.89), 0.115 |
| - None | - 114 | - 32 (29) | - 1 | - 48 (43) | - 1 | - 44 (39) | - 1 |
| - 1 | - 66 | - 36 (55) | - 1.60 (0.51), 0.138 | - 38 (58) | - 2.08 (0.69), 0.026 | - 33 (50) | - 3.59 (1.23), 0.001 |
| - 2 | - 38 | - 17 (46) | - 1.44 (0.55), 0.348 | - 19 (51) | - 1.54 (0.61), 0.274 | - 18 (49) | - 2.36 (0.96), 0.035 |
| - 3 | - 10 | - 4 (40) | - 1.56 (1.05), 0.506 | - 6 (60) | - 2.52 (1.75), 0.184 | - 5 (50) | - 2.09 (1.47), 0.295 |

- ^a^ Child emotional abuse ^b^ Child sexual abuse ^c^ Child physical abuse
- Supplementary material
- Table 7: Association of exposure to childhood emotional abuse (CEA), childhood sexual abuse (CSA) or both on mediators

| - Type of abuse | - Intrusive trauma memory   B (SE), p | - Post-traumatic avoidance and numbing - B (SE), p | - Post-traumatic hyperarousal   B (SE), p | - Negative-other beliefs   B (SE), p |
| --- | --- | --- | --- | --- |
| - CEA^a^ | - 1.12 (0.77), 0.148 | - 1.25 (0.94), 0.190 | - 0.60 (0.75), 0.426 | - 3.46 (1.34), 0.011 |
| - CSA^b^ | - 1.71 (0.93), 0.068 | - 4.03 (1.13), 0.001 | - 1.89 (0.90), 0.039 | - 3.03 (1.69), 0.074 |
| - CEA^a^ & CSA^b^ | - 1.62 (1.06), 0.128 | - 1.15 (1.29), 0.374 | - 2.12 (1.03), 0.042 | - 4.31 (1.90), 0.024 |

- ^a^ Child emotional abuse ^b^ Child sexual abuse
